# Perceived Gaps in Academic Training and Expectations From Refresher Training in Primary Care Nurses at an Urban Metropolis in Northern India: A Qualitative Study

**DOI:** 10.7759/cureus.46855

**Published:** 2023-10-11

**Authors:** Saurav Basu, Subhralaxmi Dwivedy, Jyoti Sharma, Neha Mohan, Preeti Negandhi, Shalini Goel, Mehak Gupta, Sanjay Zodpey

**Affiliations:** 1 Community Medicine, Indian Institute of Public Health-Delhi, Public Health Foundation of India, New Delhi, IND; 2 Public Health, Indian Institute of Public Health-Delhi, Public Health Foundation of India, New Delhi, IND; 3 Nutrition, Indian Institute of Public Health-Delhi, Public Health Foundation of India, New Delhi, IND; 4 Public Health, National Health Mission, Gurugram, IND; 5 Community Medicine, Public Health Foundation of India, New Delhi, IND

**Keywords:** primary care, healthcare, pedagogy, training, nursing

## Abstract

Background

Nursing professionals, comprising the largest workforce engaged in the primary healthcare system, play a pivotal role in addressing population health needs. However, gaps in the training of nurses and midwives in lower-middle-income countries may undermine their performance and necessary skill development for fulfilling key population health needs. Substantial challenges exist in improving the regular curricular and refresher training of diplomate nurses and midwives working in primary care facilities and supporting both clinical care and health promotion functions. The study objective was to conduct a gap analysis in the present nursing curriculum and training profile of general duty midwives working in urban primary health facilities and understand their expectations and preferences from the planned refresher training course.

Methods

We conducted a qualitative explorative study among General Nursing midwives (GNMs) working in urban primary health facilities in the Gurugram district of Haryana, India to conduct a gap analysis in their present curriculum and training preferences.

Results

A total of 17 nurses with a mean (SD) age of 33.52 (4.75) years and an average nursing work experience of 5.35 (0.56) years were interviewed in-depth. Lack of practical applicability, complex study material, inexperienced tutors, and weak English language comprehension were key barriers in the existing nursing curriculum. The nurses expressed willingness to participate in refresher training with varied expectations, although there existed a distinct preference for short, flexible, and blended online-offline modes of training.

Conclusions

Strengthening GNM nursing education should be prioritized in Indian health settings with the focus on improving student comprehension through vernacular instruction when feasible, and capacity building of tutors, with avenues for continued training and education. There is also a need for strengthening the curriculum related to key emergent public health challenges related to non-communicable diseases and mental health, as also skills for client and patient counseling and communication.

## Introduction

Nurses and midwives drive primary health system functioning in the developing world, especially for the provision of key sexual, reproductive, maternal, and child health services [1]. In recent years, the roles of primary care nurses have expanded in terms of establishing a continuum of care for chronic diseases including health education and behavior change communication, screening, and assessment for non-communicable diseases (NCDs) and nutritional disorders, and implementation of public health interventions including in government health programs [2]. Nursing professionals, comprising the largest workforce engaged in the primary healthcare system can therefore play a pivotal role in addressing the broader determinants of health and the interrelated aspects that influence people’s physical, mental, and social wellbeing [3,4].

In India, the country with the largest maternal and birth cohort, nurses and midwives include the community-level functionaries auxiliary nurses and midwives (ANMs), general nurses and midwives, graduate nurses, and nurses with postgraduate and diploma in specialties [3,5].

Gaps in the training of nurses and midwives undermine their performance and necessary skill development in fulfilling key population health needs [3,6,7]. Historically, it was believed that suboptimal healthcare performance was linked to inadequate education and deficient technical skills among healthcare professionals [8]. Therefore, the bulk of interventions to enhance the performance of health workers have concentrated on didacticism-based teaching, training, and dissemination of evidence-based recommendations. However, the long-term effects of such a conventional strategy have been conflicting, if not unsatisfactory [9]. An assessment of health worker performance in low-resource settings indicated that, without additional post-training support interventions, the distribution of written materials and recommendations alone-often through in-service training courses-was mostly ineffective in raising performance and quality [10].

Health worker performance and quality of care support and improvement interventions primarily include "supervision," "mentoring," and "quality improvement" strategies [11]. The International Confederation of Midwives (ICM) and the World Health Organization (WHO) have set up international standards for midwifery education and capacity building [12]. However, these interventions are frequently ineffectual in resource-limited settings of LMICs wherein primary care workers often work unsupervised in remote areas with high patient loads that overwhelm public health systems [1]. Furthermore, in India, there is a serious deficit of nurses and midwives compared to international standards [13] at 0.5 per 1000 population which is quite low compared to high-income countries (7.1 per 1000 population) and other Southeast Asian region countries (1.5 per 1000 population) [3,14]. There is pronounced regional variation in the availability of ANMs ranging from 0.7 in Bihar and Telangana to 26.6 in Andhra Pradesh suggestive of a correlation with the health system performance [15,16]. Also, there are few regulations governing nursing, midwifery coexists with nursing, and the private health industry is responsible for 88% of nursing and midwifery education, which accentuates the challenge of deficiencies in regular curricular training [17,18]. Moreover, nurses and midwives are involved in additional task load in primary health facilities to cater to the need for expanded service provisions that necessitate their further education, regulation, and association [19].

The study objective was to conduct a gap analysis in the present nursing curriculum and training profile of general duty midwives working in urban primary health facilities and understand their expectations and preferences from the planned refresher training course. 

## Materials and methods

Study design, setting, and participants

We conducted a qualitative explorative study among GNMs in the Gurugram district of the Haryana state of India. All the participants were GNMs who had completed a two-year full-time diploma course in nursing and were working as staff nurses in urban primary healthcare centers (UPHCs) with over two years of nursing work experience. In contrast to the BSc (Nursing) course which is of four-year duration and requires minimum eligibility of a higher secondary education equivalent to 12 years of schooling, the GNM nursing course has flexible and reduced schooling requirements, and the curriculum is restricted compared to the BSc (Nursing) course. The duration of the GNM course is usually three years duration apart from six months of clinical training.

Sampling procedure

A total of 23 UPHCs were present in the Gurugram district, each of which had a GNM nurse stationed, two of whom were on maternity leave. Consecutive recruitment of GNMs in the study was continued until data saturation was achieved.

Data collection procedure, management, and analysis

The participants were interviewed face-to-face by trained researchers in the Hindi language using predesigned and pretested in-depth interview (IDI) guides (Appendix). The IDI guide comprised in questions along with probes and prompts to explore the gaps they experience in their curriculum and their willingness to undertake refresher courses. The tool was validated by sourcing expert opinion from two senior public health experts with significant experience and prior involvement in the training of human resources for primary healthcare.

The interviews were audio-recorded followed by verbatim transcription and translation into English which were cross-verified by another researcher to avoid any bias in interpretation. Data were analyzed using the qualitative thematic analysis method. MAXQDA (VERBI Software, Berlin, Germany) software was used for analyzing the transcripts and generating the codes. Codes were organized, and categories and themes were generated.

Ethical consideration

The study was approved by the institutional ethics committee. Written and informed consent was obtained from all participants.

## Results

Participant characteristics

A total of 17 participants having a mean (SD) age of 33.52 (4.75) (range 27-42) years were recruited in the study. The average duration of their work experience as a nursing professional was 5.35 (±0.56) years. All the participants had done their GNM course along with post-basic nursing and all were posted in UPHCs of Gurugram. The existing GNM activities were predominantly focused on antenatal assessment, vaccination (routine immunization) of children and pregnant women, contraception services, management of minor injuries or trauma, delivery in selected PHCs, and general counseling for the health promotion of patients, caregivers, and attendants visiting their health facilities.

**Figure 1:**
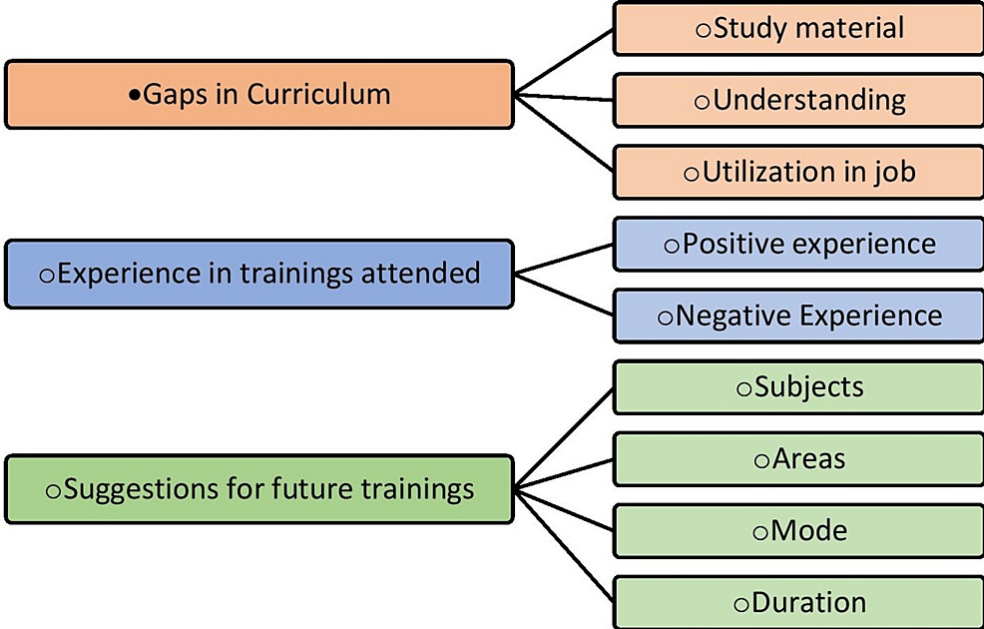
Emerged Categories and Codes

The findings were categorized under three themes: (1) Gaps in the existing nursing (GNM) curriculum, (2) Experience in undergone training (both positive and negative), and (3) Suggested pedagogical approach to refresher training (Figure 1).

Gaps in the Existing (GNM) Nursing Curriculum

One in three participants perceived that their existing course curriculum was predominantly theoretical knowledge driven lacking practical applicability in their current routine work. Some participants (n=6) reported forgetting most of their nursing coursework. One participant mentioned that:

“It has been so many years that I don’t even remember all the subjects. I can only name a few. Many of them were very theoretical. I have never even used them after I joined my work. That’s why I can’t remember them (laughs embarrassedly)”. (P3)

Gaps in the quality of nursing education were pointed out by a participant.

“I was in the first batch of the GNM course in my college. It was highly unorganized. The classes were also held in a very unorganized way. Many times, classes were not even properly held. It was like we were the guinea pigs for experimentation.” (P5)

Further, some participants confided that they could not have a proper understanding of some subjects because of the perceived complexity of the study material or inadequacy of the tutor resulting in a lack of confidence in those subjects.

“I found some of the subjects to be very difficult, especially psychiatry. I remember that I could not understand the subject and felt very dumb in class. The teacher who taught us was not very good. The study material that was provided to us seemed very difficult. Once, I discussed it with some friends of mine and I got to know that everyone was in the same boat.” (P1)

“The material which was given to us for subjects like psychiatry was incomprehensible. That led to a lack of understanding of the subject. From then on, I have never liked the subject and have always been running away from it.” (P7)

Experience in Work Training

Positive experiences: Participants very enthusiastically narrated that they look forward to the training sessions which include a doubt-clearing session, as they get to learn the most in that session. According to them, doubt-clearing sessions bring life into the training session. They undergo training conducted by the Ministry of Health and Family Welfare, Government of India at regular intervals. Trainings inclusive of doubt-clearing sessions were perceived as more effective.

“Some training sessions have a doubt-clearing session which I wait for because if there is no doubt-clearing session, it makes no sense to have training. I learn not only from my doubts but from others’ doubts also. The doubt-clearing sessions are the most fruitful parts.” (P11)

Negative experiences: Participants talked about their negative experiences with long training sessions, which didn’t contribute much to their learning.

“Sometimes training becomes very monotonous and we get bored. We are there just for the sake of it, like physically present and mentally absent.” (P3)

Participants emphasized that the gap between the training session and applicability in the work is very long and that makes the outcomes of the training sessions less effective.

“Training sessions are held when the government. The order comes and we are nominated. But most of the time, we are not using the lesson immediately or in the near future. When the time comes to use it, we have already forgotten what we learned in the training program.” (P17)

Suggested Pedagogical Approach to Refresher Training

Participants suggested avoiding information overload during the training sessions. Additionally, they felt the need to impart soft skills like communication and counseling with their expanding and dynamic roles.

“We are bombarded with information, either in classes or training sessions. We are not taught or trained about how to counsel people or convince them to practice certain methods or adapt to certain habits. More emphasis should be given to that.” (P12)

Participants proposed to have training sessions in emergent domains like mental health due to the growing help-seeking behavior of people from their communities. Lack of training in mental health was a perceived barrier in nurses offering help, referral support, and enabling their participation in mental health promotion-related activities. Moreover, the participants preferred sessions for the management of workload, frustration, and burnout at work to improve their work efficiency while minimizing stress.

“The demand for mental health has increased, especially in post COVID-19 era. If not clinical treatment, we should be trained on how to counsel the people seeking help or direct them to proper channels. This will also help the community to lead a better and happier life.” (P4)

“The workload has become too much for us, especially after COVID(pandemic), with all the testing, screening, and vaccinations going on. There are so many programs implemented by the govt. also. We also have to maintain a lot more records than we used to. This gives rise to frustration and burnout. It would be very helpful if we are trained about managing our work and having a balance.” (P6)

Some participants emphasized conducting modules related to the prevention and management of NCDs. Although the nurses had received prior refresher training on NCDs to fulfill their mandate as per the national program especially related to community screening, they perceived such training to be inadequate in effectively dealing with challenges related to raising awareness for screening and supporting the continuum of care in existing patients with NCDs.

“We have received training on different NCDs like hypertension, diabetes, cancer, etc. but I always feel that the training is not enough. It should be more detailed, and more focus should be on counseling the patients and adherence to medicine and care.” (P11)

“We need more training on the behavior change of the patients so that they can be motivated to test themselves regularly and self-assess themselves. The patients should be able to understand the intensity of the disease and how to take care of themselves. We need to be the facilitators to make them do that.” (P3)

The participant expectations from refresher training were for brief and interactive face-to-face sessions as opposed to those that lasted the whole day. A blended mode of training inclusive of both self-paced online sessions and offline sessions was preferred, but only online sessions were perceived as unsatisfactory. There was also participant concern with regard to the language of instruction for the training, both offline and online, and most nurses preferred training in the vernacular as opposed to English due to limited comprehension, especially verbal.

“The training sessions should not be very long and monotonous. They should be interactive and for a duration of 2.5 hours per day at max.” (P4)

“We like watching videos which can be a part of training. The videos can be in Hindi or our local language so that we will derive maximum knowledge from it and pass it on. If the videos are complicated and are fully in the English language, we lose interest. Further, if we have any doubts, we can get them clarified in the face-to-face sessions.” (P10)

The participants also prioritized doubt-clearing sessions and that they should be given some time to study on their own or think about the lessons imparted before the sessions begin.

“We should be given some time to reflect on the teachings before doubt-clearing sessions.” (P9)

## Discussion

**Figure 2:**
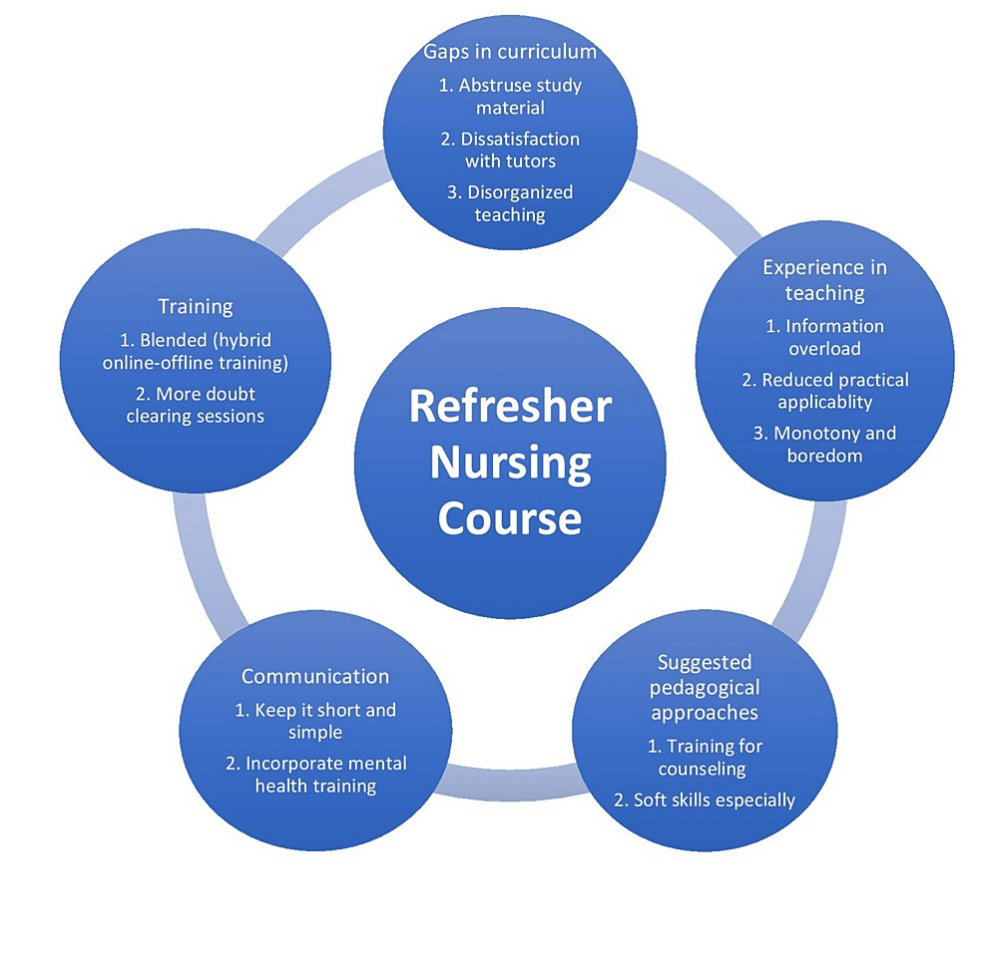
Refresher Training Course Expectations Among Nursing Staff

This qualitative study ascertained the perceived gaps in the existing academic training and identified expectations from refresher training among GNMs working in primary health centers. Our results suggest considerable heterogeneity exists in the nursing curriculum of GNM nurses with low levels of satisfaction in terms of course content, pedagogical methods, and a perceived lack of practical orientation during patient care and health promotion. While many findings implied the positive experiences of the nurses during refresher training intermittently organized by the government, issues related to insufficiency and dissatisfaction with such training also emerged. Our study indicates that vernacular medium and a blended mode of online and offline teaching with avoidance of day-long sessions and reduced intensity of coursework interspersed over a longer duration are substantially preferable as opposed to intensive short-term training (Figure 2).

India is among the 43% of LMICs to have an integrated pathway for nursing and midwifery [20]. In the present study, the participants said that the course curriculum they had was more theoretical than practical hence it is not used in their everyday work. This finding aligns with the findings of another study on capacity building of midwifery teams in Africa which reported that there do exist gaps between the theory and practice of nursing education. Furthermore, a key reason attributed to this phenomenon was the perceived deficiency in the quality of teachers which also corroborates with our study [21].

The ICM has identified regulation, education, and association as the three most important pillars for the development and practice of midwifery [22]. In India, nurses and midwives are primary healthcare providers and play an integral role in providing sexual, reproductive, and maternal healthcare [23]. 

In India, within the bachelor’s and diploma nursing courses, it is estimated that 11.6% and 18.6% of the time is allocated to midwifery, respectively; a duration, as per the WHO, not adequate to develop competent nursing midwives who could provide comprehensive maternal and newborn services [24,25]. In order to achieve its commitment to universal health coverage and strengthen its primary healthcare services, India needs to focus on improving the quality of nursing education and provide nurses with more learning opportunities to develop new skills and specializations especially those needed for improving community health standards.

Strengths and limitations

This is one of the first studies from India that assesses challenges in the curriculum of general duty midwives and understands their expectations from refresher training courses. However, there are certain study limitations. The participants included were those who had more than two years of experience therefore any new change in the curriculum in the recent year cannot be assessed. Also, the study participants were trained in a single city in Northern India, consequently, the heterogeneity of nursing education, training content, and quality across states restricts the generalizability of the study findings to other parts of the country. Finally, we did not conduct a curricular analysis of the existing GNM curriculum.

## Conclusions

This explorative qualitative study observed primary care nursing midwives working in an urban city in Northern India to experience certain dissatisfaction with their existing training curriculum. The nurse perspectives suggested the need for greater prioritization of training having practical applicability for improving routine healthcare functioning, capacity building of nursing tutors and strengthening pedagogy standards, promoting vernacular instruction when feasible, and aiding the comprehension of topics related to emergent key areas such as mental health and NCDs. Furthermore, although nurses were positively inclined to participate in refresher training for skill upgradation, a distinct preference for short, flexible, and blended online-offline mode of training over the current short duration, intense training was evident. Our findings indicate the need for a more structured evaluation of the nursing educational and training methods across India to enable pragmatic reform in enhancing the nursing and midwifery curriculum. Future studies should also assess the acceptability and efficiency of blended modes of refresher training in capacity building of primary care and public health nurses. 

## Appendices

Interview guide for GNMs

1. Please tell me about yourself. (Record Age, place of residence, marital status, work experience, education)

2. Please tell me about your course curriculum in detail. (Probes: duration of GNM course work, name of subjects, theory/practical, favorite subjects and why, neglected subjects)

 3. Is there any important subject that you think has not been taught adequately during your period of nursing education? If yes, can you please explain the reasons? (Probes: lack of staff, lack of study material, lack of understanding, no priority, lack of utilization in the job in future, any other issues) 

4. Please tell me about your work at the Urban Primary Health Centers. (Probes: when did you start, places of posting, nature of the job, like/dislike responsibilities, day-to-day activities, generalized work)

5. After you started working as a GNM, which subjects in your nursing curriculum were most applicable in your day-to-day work? (Probes: in clinical settings, in community settings)

 6. How much preventive aspect is practiced in day-to-day activities in your work? (Probes: community meetings and mobilization, maternal and child health activities, disease screening, nutrition, hygiene, mental health, tobacco risk factors, referrals, intervention, patient counseling: facilitators and barriers - knowledge-based, time-based, opportunity-based)

7. Have you received any refresher training previously? (Probes: topics (non-COVID-19 related), frequency and duration, perceived usefulness, scope for improvement) 

8. Do you think some training can be helpful to strengthen your capacities for the community? (Probes: what kind of training, benefits expected, subjects to be covered, mode of training whether online, offline, or blended) 

9. What would be your suggestion to overcome the gaps and challenges that you have faced in your training? (Probes: guidance, curricular content, specific deficiency in subject matter expertise, training for evolving roles) 
